# Supplementary material for: A yeast phenomic model for the influence of Warburg metabolism on genetic buffering of doxorubicin
Source: Cancer Metab. 2019 Oct 23;7:9. doi: 10.1186/s40170-019-0201-3 (PMC6806529; doi:10.1186/s40170-019-0201-3)
Supplement: Supplementary file 9 — Additional file 9. GO term-specific heatmaps for REMc/GTF-enriched clusters. GO term-specific heatmaps for significant GO process terms were generated as described in methods and Figs. 3 and 4. Any related child terms are presented in subsequent pages of the parent file name. GO terms with more than 100 children, with 2 or fewer genes annotated to the term, or a file size over 300KB are not shown. All heatmaps are generated with the same layout (see Figs. 3 and 4). [file 40170_2019_201_MOESM9_ESM.bz2 › Additional_File9_GOTermHeatmaps/Additional_File9_GOTermHeatmap/response_to_ionizing_radiation.pdf]

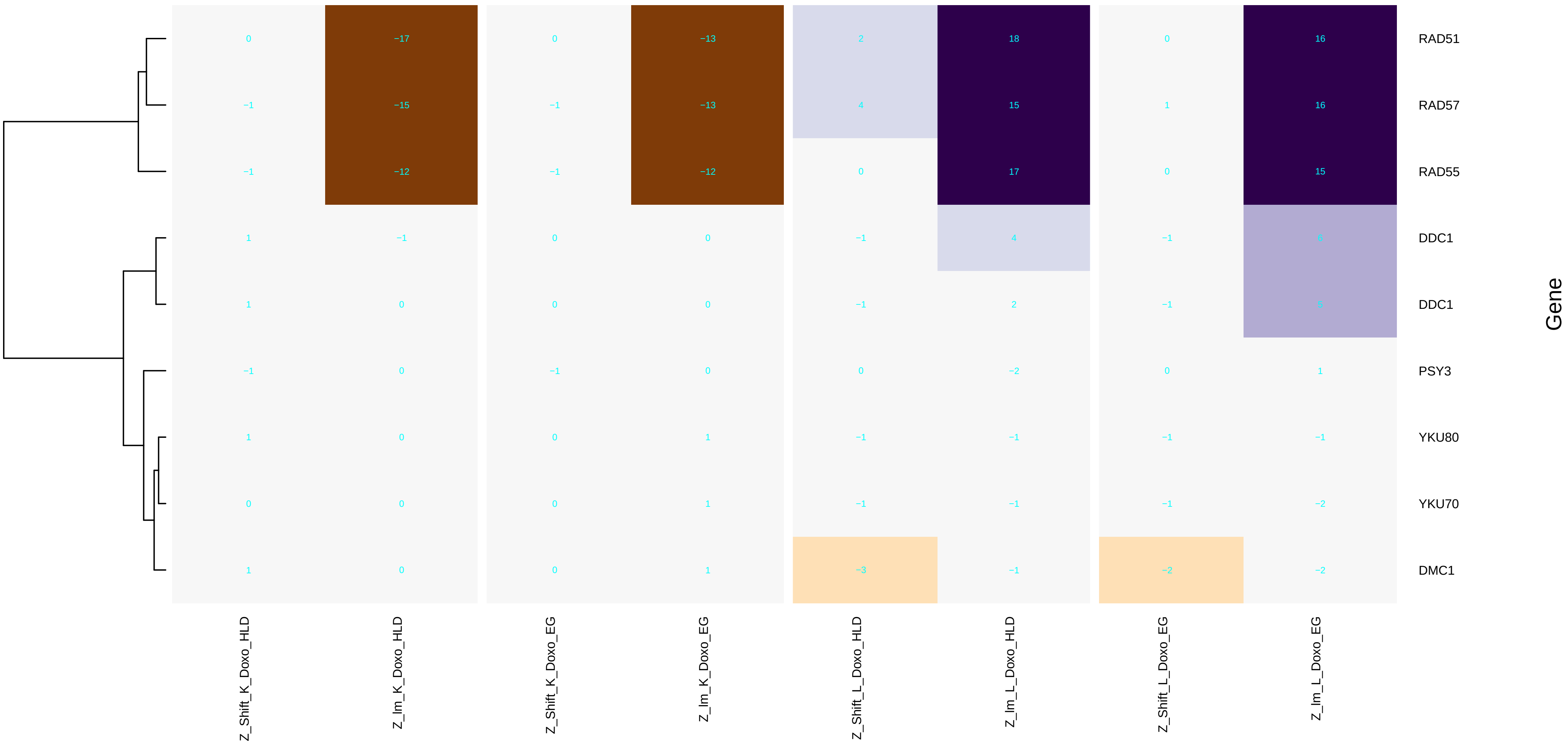

RAD51 0 −17 0 −13 2 18 0 16
RAD57 −1 −15 −1 −13 4 15 1 16
RAD55 −1 −12 −1 −12 0 17 0 15
DDC1 1 −1 0 0 −1 4 −1 6
DDC1 1 0 0 0 −1 2 −1 5
PSY3 −1 0 −1 0 0 −2 0 1
YKU80 1 0 0 1 −1 −1 −1 −1
YKU70 0 0 0 1 −1 −1 −1 −2
DMC1 1 0 0 1 −3 −1 −2 −2
Z_Shift_K_Doxo_HLD
Z_Im_K_Doxo_HLD
Z_Shift_K_Doxo_EG
Z_Im_K_Doxo_EG
Z_Shift_L_Doxo_HLD
Z_Im_L_Doxo_HLD
Z_Shift_L_Doxo_EG
Z_Im_L_Doxo_EG
Gene
